# Anemia and growth failure among HIV-infected children in India: a retrospective analysis

**DOI:** 10.1186/1471-2431-9-37

**Published:** 2009-06-16

**Authors:** Anita Shet, Saurabh Mehta, Nirmala Rajagopalan, Chitra Dinakar, Elango Ramesh, NM Samuel, CK Indumathi, Wafaie W Fawzi, Anura V Kurpad

**Affiliations:** 1 Department of Pediatrics, St John's National Academy of Health Sciences, Bangalore 560034, India; 2 Departments of Nutrition and Epidemiology, Harvard School of Public Health, Boston, MA 02115, USA; 3 Freedom Foundation, Bangalore, India; 4 ART Clinic, Namakkal Government Hospital, Namakkal, India; 5 Concern for AIDS Research and Education, Namakkal, India; 6 Division of Nutrition, St John's Research Institute, St. John's National Academy of Health Sciences, Bangalore 560034, India

## Abstract

**Background:**

Anemia and poor nutrition have been previously described as independent risk factors for death among HIV-infected children. We sought to describe nutritional status, anemia burden and HIV disease correlates among infected children in India.

**Methods:**

We analyzed retrospective data from 248 HIV-infected children aged 1–12 years attending three outpatient clinics in South India (2004–2006). Standard WHO definitions were used for anemia, HIV staging and growth parameters. Statistical analysis included chi square, t tests, univariate and multivariate logistic regression analyses.

**Results:**

The overall prevalence of anemia (defined as hemoglobin < 11 gm/dL) was 66%, and 8% had severe anemia (Hb < 7 gm/dL). The proportion of underweight and stunted children in the population was 55% and 46% respectively. Independent risk factors of anemia by multivariate analysis included the pre-school age group (age younger than 6 years) (OR: 2.87; 95% CI: 1.45, 5.70; p < 0.01), rural residence (OR: 12.04; 95% CI: 5.64, 26.00; p < 0.01), advanced HIV disease stage (OR: 6.95; 95% CI: 3.06, 15.79; p < 0.01) and presence of stunting (Height-for-age Z Score < -2) (OR: 3.24; 95% CI: 1.65, 6.35; p < 0.01). Use of iron/multivitamin supplementation was protective against risk of anemia (OR: 0.44; 95% CI: 0.22, 0.90; p = 0.03). Pulmonary tuberculosis was an independent risk factor in multivariate analysis (OR: 3.36; 95% CI: 1.43, 7.89; p < 0.01) when correlated variables such as HIV disease stage and severe immunodeficiency, and nutritional supplement use were not included. Use of antiretroviral therapy (ART) was associated with a reduced risk of anemia (OR: 0.29; 95% CI: 0.16, 0.53; p < 0.01). No significant association was found between anemia and gender, cotrimoxazole, or ART type (zidovudine versus stavudine).

**Conclusion:**

The high prevalence and strong interrelationship of anemia and poor nutrition among HIV-infected children in India, particularly those living in rural areas underscores the need for incorporating targeted nutritional interventions during national scale up of care, support and treatment among HIV-infected children.

## Background

Globally, the HIV epidemic remains a serious challenge, and continues to take its toll particularly on vulnerable populations such as children. Although revised 2007 UNAIDS epidemiological analyses indicate that the current Indian national seroprevalence is 0.36%, ongoing perinatal transmission substantially impacts the incidence of paediatric HIV, adding to the large pool of HIV-infected children in India [1]. In 2005, the National AIDS Control Organization has dramatically increased access to antiretroviral therapy for children, and several thousands of children have been successfully initiated on specific anti-HIV therapy. However, background co-morbidities compound the problem in affected populations in India. Two such major co-morbidities include anemia and poor nutrition, whose detrimental effects are magnified in the context of HIV infection.

Studies have unequivocally demonstrated that anemia is associated with decreased survival and increased disease progression in adults with HIV infection [2-4]. Independent of other factors, anemia is also associated with a diminished quality of life [5]. In children with HIV infection, the high prevalence of anemia is well known[6]. Given that the negative impact of anemia is magnified on account of its close relation to overall nutrition and growth, there is limited data from Asian countries where HIV infection, malnutrition and nutritional deficiencies co-exist. Understanding nutritional co-morbidities will be beneficial in planning appropriate intervention strategies to reduce the overall burden of pediatric HIV in India. In this report, we describe nutritional status, HIV disease stage, and correlates of anemia among HIV-infected children in South India.

## Methods

A retrospective cross-sectional, multicenter approach was used, with collation of data from medical records of HIV-infected children, aged 1 to 12 years, attending three outpatient clinics in India from 2004 to 2006. The sites included a tertiary care centre (St. John's Medical College Hospital, Bangalore), an urban community clinic (Freedom Foundation, Bangalore), and a rural community clinic (Namakkal Government Hospital, Tamil Nadu). Ethical approval from the local Institutional Review Board from the study sites was obtained prior to examination of patient records. Relevant clinical information recorded included the children's weight, height, detailed history and physical examination, baseline laboratory values such as haemoglobin, total white blood cell count and CD4+ T cell count. Specific information on use of iron supplements as recorded in the medical chart was noted. The place of residence was taken from the address given to the clinic while registering for medical treatment. Out of a possible 387 children from all sites, 248 children with all clinical points documented simultaneously were included in the final analysis, while 139 with missing height, haemoglobin, or clinical staging documentation were excluded. Children were diagnosed and classified according to clinical and immunological categories according to WHO criteria [7]. Ambulatory management of children attending these clinics included routine clinical monitoring, treatment of minor infections, cotrimoxazole prophylaxis, Anti-Retroviral Therapy (ART) administration and nutritional supplementation.

Anemia was defined as a haemoglobin concentration of < 11 g/dL, and severe anemia defined as haemoglobin of < 7 g/dL, based on the WHO description of anemia [8]. Information about the children's weight and height was retrieved from the medical records. Z scores were calculated for each child using EpiInfo Nutstat (version 3.4.3, Centers for Disease Control and Prevention, Atlanta Georgia) and used to express the standard deviation in SD units from the age- and gender-based reference growth standards developed by the National Center for Health Statistics [9,10]. Weight-for-age Z score less than -2 SD, height-for-age Z score less than -2 SD and weight-for-height Z score less than -2 SD were considered consistent with underweight, stunting and wasting respectively.

Mean and standard deviations were used to describe normally distributed data from the subjects' demographic, anthropometric and laboratory measurements. Categorical and continuous data were compared using chi-square and Kruskal-Wallis tests respectively. The Spearman rank correlation test was used to determine the relationship between different continuous variables. A p value of < 0.05 was considered significant. Odds ratios and 95% confidence intervals were estimated using univariate logistic regression analyses to evaluate the association of nutrition status, HIV clinical stage, CD4+ count, and ART with anemia. Variables with univariate p-values < 0.2 were included in a multivariate logistic regression model and retained if their p-value was < 0.05. Statistical analyses were performed using SAS software version 9.1 (SAS Institute Inc., Cary, NC, US).

## Results

### Study population

Among 248 children who were included, 57% were males, and mean age was 7 years (SD: 3.4 yrs) (Table 1). The majority of the children had no or mild symptoms of HIV infection (69% in WHO Clinical Stage 1 and 2). Advanced disease was present in 31% of children (WHO Clinical Stage 3 and 4). Thirty-four percent had no evidence of immunosuppression, 44% had mild-to-advanced immunosuppression and 22% of children were severely immunosuppressed based on the age-stratified CD4 thresholds adopted by the WHO classification[7]. ART naïve children constituted 77% of the total population, and the remaining 23% of the children had been on ART for an average period of 1.2 years. When compared to children included in the final analysis, excluded children with missing data (n = 139) had no significant differences in hemoglobin values, place of residence (urban versus rural), age and clinical stage.

**Table 1:** Characteristics of 248 HIV-infected children

**Characteristic**	**Mean (SD^b^)/Proportion**
Age, years	7.24 years (3.44)

Gender (boys)	56.45%

HIV Staging	
Stage 1 and 2	68.95%
Stage 3 and 4	31.05%
Severe immunodeficiency^a^	21.77%

CD4 count	582 cells/mm^3 ^(401)

Weight-for-age Z score (WAZ)	-1.98 (1.45)

Height-for-age Z score (HAZ)	-1.71 (1.89)

Underweight (WAZ < -2)	55.24%

Stunting (HAZ < -2)	46.37%

Wasting (weight-for-height Z score < -2)	34.27%

On antiretroviral therapy (ART)	22.58%

Hemoglobin	9.95 g/dl (1.93)

HIV/TB co-infection	21.77%

^a ^Immunodeficiency defined according to age-stratified WHO classification [7]; CD4 count < 750 for 1–3 year old children; < 350 for 3–5 year olds; and < 200 for older than 5 years.

^b^SD: Standard deviation

### Anemia in the study population

**Figure 1:**
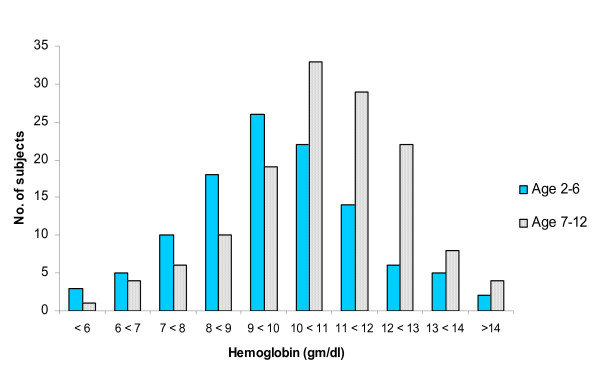
**Distribution of hemoglobin levels among children of different age groups, pre-school (1–6 yrs) and school-going (7–12 yrs)**.

The overall prevalence of anemia was 66%, and 8% had severe anemia (Hb < 7 g/dL). The mean haemoglobin was 9.95 g/dL (SD: 1.93). As shown in Figure 1, haemoglobin levels were lower in the pre-school age group, compared to the 7–12 year age group. The prevalence of anemia in clinical stages 1, 2, 3 and 4 was 51%, 58%, 87% and 72% respectively. Haemoglobin level was significantly lower among those with advanced and severe clinical stages, compared to those in stage 1 and 2 (9.2 gm/dl vs. 10.3 gm/dl; *p = 0.03*). With respect to immunological stage, anemia was present in 85% of those with advanced or severe immunosuppression while only 56% of children with mild or no immunosuppression were anemic *(p < 0.01)*.

### Risk factors and correlates for anemia

In univariate logistic regression analyses, age younger than 6 years (Table 2; Odds Ratio [OR]: 2.68; 95% Confidence Intervals [CI]: 1.55, 4.61), advanced HIV disease stage, (OR: 3.47; 95% CI: 1.83, 6.57) and severe immunodeficiency (OR: 4.5; 95% CI: 2.01, 10.0) were significantly correlated with higher odds of anemia. A rural address was more likely to be associated with the presence of anemia; among 119 children who hailed from a rural setting 83% had anemia, whereas 43% of the 129 children from an urban background had anemia *(p < 0.01)*. On the other hand, use of ART was associated with a 70% reduced odds of having anemia *(p < 0.01)*. A total of 50 children were receiving institutionalized care at the time of data recording, and anemia was found to be less prevalent in this group compared to those children living at home with their families (44% *vs*. 67%, respectively, *p < 0.01)*. Anemia was also associated with pulmonary tuberculosis; hemoglobin in those children with and without tuberculosis was 8.9 and 10.2 g/dl respectively *(p < 0.01)*. There was no significant association between prevalence of anemia with gender, use of cotrimoxazole prophylaxis, or type of antiretroviral drug (zidovudine versus stavudine).

**Table 2:** Correlates of Anemia in HIV-infected children (*n *= 248)

**Risk Factors**	**Univariate correlates**		**Multivariate correlates**^a^	
	**Odds ratio (95% CI)**	**p-value**	**Odds ratio (95%CI)**	**p-value**
				


**Socio-demographic**				

Age (Pre-school vs. school-age)^b^	2.68 (1.55, 4.61)	< 0.01	2.87 (1.45, 5.70)	< 0.01

Sex (males vs. females)	0.97 (0.58, 1.62)	0.89		

Rural vs. urban	6.45 (3.57, 11.68)	< 0.01	12.09 (5.64, 25.96)	< 0.01

				

**HIV and care-related**				

Advanced HIV stage (Stage 3,4 vs. 1,2)	3.47 (1.83, 6.57)	< 0.01	6.95 (3.06, 15.79)	< 0.01

Advanced/severe immunodeficiency^c^	4.48 (2.01, 10.0)	< 0.01		

Antiretroviral therapy (ART)	0.29 (0.16, 0.53)	< 0.01		

ART type (d4T vs. AZT)	1.35 (0.43, 4.30)	0.81		

Use of cotrimoxazole	1.15 (0.68, 1.92)	0.61		

TB/HIV co-infection	3.29 (1.57, 6.92)	< 0.01	3.36 (1.43, 7.89)	< 0.01^d^

Use of nutritional supplements (multivitamins, iron)	0.68 (0.40, 1.16)	0.15	0.44 (0.22, 0.90)	0.03

Care (institutionalized vs. home-based)	0.38 (0.20, 0.72)	< 0.01		

				

**Anthropometric**				

Underweight	2.56 (1.51, 4.34)	< 0.01		

Stunting	3.21 (1.85, 5.56)	< 0.01	3.24 (1.65, 6.35)	< 0.01

Wasting	1.58 (0.91, 2.76)	0.11		

In multivariate analyses, children of pre-school age were three times more likely to be anemic compared to children of school age (Table 2; OR: 2.87; 95% CI: 1.45, 5.70; p < 0.01). The odds of having anemia in HIV-infected children from rural areas were 12 times higher compared to children from urban areas (OR: 12.04; 95% CI: 5.64, 26.00; p < 0.01). Children with WHO stage 3 or 4 HIV disease were 7 times more likely to have anemia compared to children with WHO stage 1 or 2 HIV disease (OR: 6.95; 95% CI: 3.06, 15.79; p < 0.01). HIV-infected children with stunting had over 3 times the odds of having anemia compared to children without stunting (OR: 3.24; 95% CI: 1.65, 6.35; p < 0.01). Supplementation with iron or multivitamins was associated with 56% reduced odds of anemia (OR: 0.44; 95% CI: 0.22, 0.90; p = 0.03). In a separate model, where iron or multivitamin supplements and correlated variables such as advanced HIV disease stage and severe immunodeficiency were not included, coinfection with pulmonary tuberculosis was also associated with higher odds of anemia (OR: 3.36; 95% CI: 1.43, 7.89; p < 0.01).

### Prevalence of growth failure

The proportion of underweight and stunted children in the population was 55% and 46% respectively (Table 1). Only 34% of children were wasted (weight-for-height Z score < -2) indicating that proportional growth failure is more likely in the setting of HIV infection rather than acute weight loss over a short period. Being underweight (weight-for-age Z score < -2) was associated with advanced HIV disease stage (*p = 0.019*) while stunting (*p = 0.06*) and wasting (*p = 0.5*) did not correlate with clinical stage, suggesting that cachexia and disproportionate weight loss are the more typical pattern of advanced disease progression in HIV-infected children. Children on at least 6 months of ART were less likely to be underweight compared to those who were ART-naïve (38% versus 60%; *p = 0.002*).

### Prevalence of anemia by nutritional status

**Figure 2:**
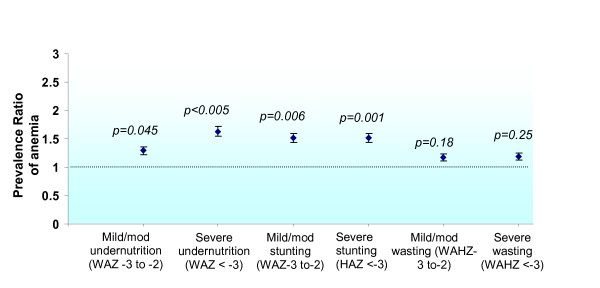
**Prevalence Ratio (PR) for anemia by categories of malnutrition (underweight, stunting, wasting)**. Anemia is significantly associated with the underweight state and with stunting, but not with wasting. WAZ: weight for age Z score; HAZ: height for age Z score; WAHZ: weight-for-height Z score.

Anemia was significantly associated with poor growth (WAZ, HAZ < -2) and advanced HIV disease status (p < 0.005). To examine anemia prevalence by different categories of malnutrition, children were stratified into those with mild or moderate (Z scores -3 to -2) versus severe (Z scores < -3) degrees of being underweight, stunted or wasted. The reference population for this analysis was the group of children with no malnutrition (Figure 2). The prevalence ratios (PR) of anemia among the various categories were as follows: mild-to-moderate underweight status: 1.29 (95% CI: 1.01, 1.65); severe underweight status: 1.63 (95% CI: 1.31, 2.04); mild-to-moderate stunting: 1.52 (95% CI: 1.22, 1.89); severe stunting: 1.52 (95% CI: 1.21, 1.9); mild-to-moderate wasting: 1.17 (95% CI: 0.94, 1.46) and severe wasting: 1.19 (95% CI: 0.91, 1.55). Notably, there was a lack of association between anemia prevalence and wasting.

## Discussion

As with previous studies from resource-limited settings [11-17], our data suggest that there is a high prevalence of anemia and growth failure among children living with HIV infection in India. Both anemia and growth failure were associated with advanced HIV disease among children in this report. In a meticulously conducted review on the global prevalence of HIV-associated anemia, Calis *et al *reported that anemia was a common complication occurring in 50–90% of children living with HIV in both resource-limited and resource-rich settings and that anemia prevalence was over three times higher among these children when compared with those without HIV infection[6]. Recent longitudinal studies show that anemia is an independent predictor of mortality among children with HIV infection[18].

Children in the pre-school age group are at considerably higher risk for developing anemia, possibly due to their increased growth requirements and higher frequency of gastrointestinal infections. The vulnerability of this age group and heightened propensity for developing anemia has been consistently reported in other studies [19]. This point has particular relevance in practice and policy implications as interventions targeted toward younger age groups are likely to have maximum benefits. The increased vulnerability of HIV-infected children hailing from a rural setting for developing anemia was highlighted in our report. Increased anemia prevalence among rural children has been demonstrated within the general population, as in the recent National Family Health Survey-3 results within India [20], and is often attributed to diminished quality of nutritional intake. National reports also indicate that the rural population has higher HIV prevalence than the urban population [21]. Taken together, nutritional interventions aimed at improving quality of life among HIV-infected children should include strategies relevant in rural settings in India.

Not surprisingly, our data indicate that anemia was more severe among those with advanced disease stage and a higher degree of immunosuppression. Both adult [2-4] and paediatric studies [11,12,15,22,23] have demonstrated a higher anemia prevalence among those with more advanced disease. A haemoglobin value of < 8 gm/dL that is unresponsive to non-ART treatment has been included as a criterion for WHO Stage 3 clinical classification, and is considered as adequate criterion for initiating antiretroviral therapy [24]. Advanced HIV disease may be associated with deficiencies of other micronutrients in children; such as vitamin A which is thought to have a role in erythropoiesis and iron transport [25] The presence of opportunistic infection among those with advanced disease can further compound anemia. The finding that HIV-infected children with pulmonary tuberculosis were three times more likely to have anemia is consistent with other reports that indicate that co-infection with tuberculosis is a potent risk factor for anemia, particularly severe anemia [26]. Another potential contributing factor is anemia of chronic inflammation where there is hepcidin-mediated decreased intestinal iron absorption and increased conservation of iron within the reticuloendothelial system resulting in decreased erythropoiesis [27].

The myriad etiologies of anemia among children with HIV have been well summarized previously [6]. Common causes include nutrient deficiencies, HIV-mediated suppression of erythropoiesis, drug effects, other opportunistic infections and HIV-associated malignancies. In resource-limited settings, nutritional deficiencies abound, especially iron, folic acid, zinc and vitamin A deficiencies[28]. Iron deficiency is particularly interesting in this population; although frequently reported in HIV-infected subjects, studies with an HIV-uninfected control group did not clearly suggest increased prevalence of iron deficiency among HIV-infected children in comparison to uninfected children [11,29]. Observational studies have raised concerns that iron supplementation may have adverse consequences in HIV infection [30-33]. Further studies are needed in order to unravel the questions on iron and HIV infection.

There is limited data analysing the effect of pre-existing anemia on disease progression among children with HIV infection who are also at high risk for nutritional deficiencies [34]. In multivariate analysis, our data indicated that receipt of nutritional supplements was significantly and independently associated with decreased odds of being anemic. A randomized controlled study from Tanzania reported that women who took multivitamin supplementation during pregnancy and postpartum period showed improved hematologic status; a beneficial effect was also seen among their children who were less likely to have microcytic hypochromic anemia [35]. The improved hematologic status among children may have contributed to a better clinical outcome. It is conceivable that treatment of anemia and provision of nutritional supplements in HIV-infected children will have a beneficial effect independent of antiretroviral therapy in improving morbidity and mortality, although this has not been unequivocally demonstrated among children living in resource limited settings[36,37].

In general, poor growth is reported in as many as 50% of HIV-infected children [16]. The prevalence of malnutrition among HIV-infected children reported from Indian studies has a wide range (17% to 62%) owing to differing definitions [38-40]. Growth failure may be a direct consequence of the HIV infection, secondary to the clinical illness associated with HIV, a function of the child's adverse environment, or a combination of these factors. It is probable that, independent of HIV infection, malnutrition can reduce immunological function and can impair that child's ability to resolve acute infections. Low weight for age, like low haemoglobin, was an independent predictor of mortality among HIV-infected children in Zambia[16,34]. The pattern of growth failure among the children in this study suggested the prevalence of both relatively acute (underweight or low weight-for-age) and chronic growth failure (stunted or low height-for-age). The overall prevalence of wasting (low weight-for-height) was low, as seen in our study, suggesting that the majority of children with growth failure as indicated by underweight or stunting, were nevertheless, normally proportioned. Importantly, our data also indicated that HIV-infected children who were underweight and stunted were also more likely to be anemic. In particular, multivariate analyses indicated that stunting was independently associated with increased prevalence of anemia in this cohort. The beneficial effect of ART on anemia and growth parameters has been demonstrated previously [41,42]. A large longitudinal analysis from South India that included a cohort of 295 children (mean age, 7.6 years; median baseline CD4 percentage, 14%) who were newly initiated on ART reported that baseline prevalence of anemia (Hb < 11 gm/dL) was 66% and that of severe malnutrition (weight-for-age Z score < -2) was 35% [43]. Within just 6 months of ART initiation, a significant increase in haemoglobin and weight gain (1.6 gm/dL and 2 kg, respectively) was noted [43]. As the analyses reported here represent cross-sectional data, a clear improvement was not demonstrated in this cohort. However, the finding that provision of nutritional supplements is independently associated with better health parameters needs to be explored further.

The retrospective design of the study precluded the use of concomitant HIV-negative controls. In comparison with non-infected children, HIV-infected children are likely to have increased nutritional requirements, poor appetite and reduced intake due to illness and socioeconomic factors [16]. Anemia is also likely to be more prevalent among HIV-infected children compared to the general population and may be attributed to its multifactorial nature. Nutritional deficiencies may be magnified in this setting, opportunistic infections and parasitic infestations may result in increased losses, and the presence of chronic infection may depress erythropoeisis [6]. The WHO recommends a 10% increase in energy intake for asymptomatic HIV-infected children, with further increases of 20–30% and 50–100% for those children who are symptomatic and experiencing weight loss respectively[44]. There are no specific recommendations with respect to iron supplementation in HIV-infected children. Observational studies may show benefit of iron and other nutritional supplements for HIV-infected populations; however no clear evidence is present to base recommendations of iron supplementation in HIV-infected children [45].

The retrospective nature of the study limited our understanding of the extent to which ART and nutritional interventions can improve overall nutritional status among children. Children with missing data were not included in the analyses, hence the prevalence and correlations reported in this study reflect only a proportion of all the HIV-infected children in this region. Study limitations also included the non-standardization of haemoglobin estimation methods used across sites; two of the sites used an automated haematology analyzer while the third site used the manual Sahli's method. Additionally, the specific etiology of anemia and growth failure has not been addressed in this study. Despite these limitations, this study yields important epidemiological information on anemia and growth failure among children with HIV infection in India, and highlights the need for further large scale prospective studies to understand the scale and etiology of the problem so as to plan appropriate interventions.

## Conclusion

Our study reinforces the finding that anemia, growth failure, and malnutrition are major manifestations of HIV infection in Indian children with prognostic significance. Identification of major independent risk factors for anemia such as age younger than 6 years, rural residence and co-infection with pulmonary tuberculosis can guide appropriate therapeutic interventions. Our data highlights the beneficial effect of antiretroviral therapy and iron/multivitamin supplementation in reducing risk of anemia. In addition to continuing efforts to improving access to antiretroviral therapy, it is time to pay attention to further refining our therapeutic strategies by making nutritional counselling, nutrition supplementation and sustainable nutritional interventions an integral part of our overall approach to helping children with HIV live as normal a life as possible.

## Competing interests

The authors declare that they have no competing interests.

## Authors' contributions

AS conceived of the study, and participated in its design, data analysis and interpretation and drafted the manuscript. SM helped conduct the statistical analysis. SM and WF helped with interpretation of results and contributed towards drafting and review of manuscript. NR participated in data acquisition and manuscript review. AVK provided critical review of manuscript. CD, ER, NMS, and CKI participated in data acquisition. All authors have read and have approved the final manuscript.

## Pre-publication history

The pre-publication history for this paper can be accessed here:



## References

[B1] AIDS Epidemic Update, Dec 2007. UNAIDS Report. http://data.unaids.org/pub/EPISlides/2007/2007_epiupdate_en.pdf.

[B2] Mocroft A, Kirk O, Barton SE, Dietrich M, Proenca R, Colebunders R, Pradier C, dArminio Monforte A, Ledergerber B, Lundgren JD (1999). Anaemia is an independent predictive marker for clinical prognosis in HIV-infected patients from across Europe. EuroSIDA study group. AIDS.

[B3] Moore RD, Keruly JC, Chaisson RE (1998). Anemia and survival in HIV infection. J Acquir Immune Defic Syndr Hum Retrovirol.

[B4] Sullivan PS, Hanson DL, Chu SY, Jones JL, Ward JW (1998). Epidemiology of anemia in human immunodeficiency virus (HIV)-infected persons: results from the multistate adult and adolescent spectrum of HIV disease surveillance project. Blood.

[B5] Volberding PA, Levine AM, Dieterich D, Mildvan D, Mitsuyasu R, Saag M (2004). Anemia in HIV infection: clinical impact and evidence-based management strategies. Clin Infect Dis.

[B6] Calis JC, van Hensbroek MB, de Haan RJ, Moons P, Brabin BJ, Bates I (2008). HIV-associated anemia in children: a systematic review from a global perspective. AIDS.

[B7] (2006). WHO case definitions of HIV for surveillance and revised clinical staging and immunological classification of HIV-related disease in adults and children.

[B8] (2001). Iron deficiency anaemia: assessment, prevention, and control. A guide for program managers.

[B9] National Center for Health Statistics 2000 CDC Growth Charts. http://www.cdc.gov/growthcharts/.

[B10] Kuczmarski RJ, Ogden CL, Guo SS, Grummer-Strawn LM, Flegal KM, Mei Z, Wei R, Curtin LR, Roche AF, Johnson CL (2002). 2000 CDC Growth Charts for the United States: methods and development. Vital Health Stat 11.

[B11] Totin D, Ndugwa C, Mmiro F, Perry RT, Jackson JB, Semba RD (2002). Iron deficiency anemia is highly prevalent among human immunodeficiency virus-infected and uninfected infants in Uganda. J Nutr.

[B12] Eley BS, Sive AA, Shuttleworth M, Hussey GD (2002). A prospective, cross-sectional study of anaemia and peripheral iron status in antiretroviral naive, HIV-1 infected children in Cape Town, South Africa. BMC Infect Dis.

[B13] Clark TD, Mmiro F, Ndugwa C, Perry RT, Jackson JB, Melikian G, Semba RD (2002). Risk factors and cumulative incidence of anaemia among human immunodeficiency virus-infected children in Uganda. Ann Trop Paediatr.

[B14] Semba RD, Broadhead R, Taha TE, Totin D, Ricks MO, Kumwenda N (2001). Erythropoietin response to anemia among human immunodeficiency virus-infected infants in Malawi. Haematologica.

[B15] Adewuyi J, Chitsike I (1994). Haematologic features of the human immunodeficiency virus (HIV) infection in Black children in Harare. Cent Afr J Med.

[B16] Arpadi SM (2000). Growth failure in children with HIV infection. J Acquir Immune Defic Syndr.

[B17] Bailey RC, Kamenga MC, Nsuami MJ, Nieburg P, St Louis ME (1999). Growth of children according to maternal and child HIV, immunological and disease characteristics: a prospective cohort study in Kinshasa, Democratic Republic of Congo. Int J Epidemiol.

[B18] (2008). Markers for predicting mortality in untreated HIV-infected children in resource-limited settings: a meta-analysis. AIDS.

[B19] Calis JC, Phiri KS, Faragher EB, Brabin BJ, Bates I, Cuevas LE, de Haan RJ, Phiri AI, Malange P, Khoka M (2008). Severe anemia in Malawian children. N Engl J Med.

[B20] (2007). National Family Health Survey (NFHS-3) 2005–06: Key Findings.

[B21] Kumar R, Jha P, Arora P, Mony P, Bhatia P, Millson P, Dhingra N, Bhattacharya M, Remis RS, Nagelkerke N (2006). Trends in HIV-1 in young adults in south India from 2000 to 2004: a prevalence study. Lancet.

[B22] Ellaurie M, Burns ER, Rubinstein A (1990). Hematologic manifestations in pediatric HIV infection: severe anemia as a prognostic factor. Am J Pediatr Hematol Oncol.

[B23] Adetifa IM, Temiye EO, Akinsulie AO, Ezeaka VC, Iroha EO (2006). Haematological abnormalities associated with paediatric HIV/AIDS in Lagos. Ann Trop Paediatr.

[B24] Antiretroviral therapy for HIV infection in adults and adolescents (2006). Recommendations for a public health approach 2006.

[B25] Zimmermann MB, Biebinger R, Rohner F, Dib A, Zeder C, Hurrell RF, Chaouki N (2006). Vitamin A supplementation in children with poor vitamin A and iron status increases erythropoietin and hemoglobin concentrations without changing total body iron. Am J Clin Nutr.

[B26] Swaminathan S, Padmapriyadarsini C, Sukumar B, Iliayas S, Kumar SR, Triveni C, Gomathy P, Thomas B, Mathew M, Narayanan PR (2008). Nutritional status of persons with HIV infection, persons with HIV infection and tuberculosis, and HIV-negative individuals from southern India. Clin Infect Dis.

[B27] Andrews NC (2004). Anemia of inflammation: the cytokine-hepcidin link. J Clin Invest.

[B28] Eley BS, Sive AA, Abelse L, Kossew G, Cooper M, Hussey GD (2002). Growth and micronutrient disturbances in stable, HIV-infected children in Cape Town. Ann Trop Paediatr.

[B29] Miller MF, Humphrey JH, Iliff PJ, Malaba LC, Mbuya NV, Stoltzfus RJ (2006). Neonatal erythropoiesis and subsequent anemia in HIV-positive and HIV-negative Zimbabwean babies during the first year of life: a longitudinal study. BMC Infect Dis.

[B30] Salmon-Ceron D, Fontbonne A, Saba J, May T, Raffi F, Chidiac C, Patey O, Aboulker JP, Schwartz D, Vilde JL (1995). Lower survival in AIDS patients receiving dapsone compared with aerosolized pentamidine for secondary prophylaxis of Pneumocystis carinii pneumonia. Study Group. J Infect Dis.

[B31] Jacobus DP (1996). Randomization to iron supplementation of patients with advanced human immunodeficiency virus disease – an inadvertent but controlled study with results important for patient care. J Infect Dis.

[B32] Salhi Y, Costagliola D, Rebulla P, Dessi C, Karagiorga M, Lena-Russo D, de Montalembert M, Girot R (1998). Serum ferritin, desferrioxamine, and evolution of HIV-1 infection in thalassemic patients. J Acquir Immune Defic Syndr Hum Retrovirol.

[B33] de Monye C, Karcher DS, Boelaert JR, Gordeuk VR (1999). Bone marrow macrophage iron grade and survival of HIV-seropositive patients. AIDS.

[B34] Walker AS, Mulenga V, Sinyinza F, Lishimpi K, Nunn A, Chintu C, Gibb DM (2006). Determinants of survival without antiretroviral therapy after infancy in HIV-1-infected Zambian children in the CHAP Trial. J Acquir Immune Defic Syndr.

[B35] Fawzi WW, Msamanga GI, Kupka R, Spiegelman D, Villamor E, Mugusi F, Wei R, Hunter D (2007). Multivitamin supplementation improves hematologic status in HIV-infected women and their children in Tanzania. Am J Clin Nutr.

[B36] Irlam JH, Visser ME, Rollins N, Siegfried N (2005). Micronutrient supplementation in children and adults with HIV infection. Cochrane Database Syst Rev.

[B37] Friis H (2006). Micronutrient interventions and HIV infection: a review of current evidence. Trop Med Int Health.

[B38] Merchant RH, Oswal JS, Bhagwat RV, Karkare J (2001). Clinical profile of HIV infection. Indian Pediatr.

[B39] Shah SR, Tullu MS, Kamat JR (2005). Clinical profile of pediatric HIV infection from India. Arch Med Res.

[B40] Lodha R, Upadhyay A, Kabra SK (2005). Antiretroviral Therapy in HIV-1 Infected Children. Indian Pediatr.

[B41] Kabue MM, Kekitiinwa A, Maganda A, Risser JM, Chan W, Kline MW (2008). Growth in HIV-infected children receiving antiretroviral therapy at a pediatric infectious diseases clinic in Uganda. AIDS Patient Care STDS.

[B42] Nachman SA, Lindsey JC, Moye J, Stanley KE, Johnson GM, Krogstad PA, Wiznia AA (2005). Growth of human immunodeficiency virus-infected children receiving highly active antiretroviral therapy. Pediatr Infect Dis J.

[B43] Rajasekaran S, Jeyaseelan L, Ravichandran N, Gomathi C, Thara F, Chandrasekar C (2008). Efficacy of Antiretroviral Therapy Program in Children in India: Prognostic Factors and Survival Analysis. J Trop Pediatr.

[B44] Nutrient requirements for people living with HIV/AIDS: report of a technical consultation, World Health Organization, Geneva, 13–15 May 2003.

[B45] Adetifa I, Okomo U (2009). Iron supplementation for reducing morbidity and mortality in children with HIV. Cochrane Database Syst Rev.

